# Whole Wheat Bread

**Published:** 1892-05

**Authors:** 


### WHOLE WHEAT BREAD.

As milk contains all the elements of nutrition necessary for a child, so wheat bread and its congeners, oats, rye, etc., may be considered the representatives of all human food, as these cereals contain all the elements for the nutrition of the body in every stage of life, and under all circumstances of climate, occupation, etc. But this is true only of the entire grain, and not of the fine white flour after the grain has been ground and carried through the bolting cloth. In this process the grain is well nigh deprived of all its nutritive properties, and its value as a food sacrificed to the whim of having white bread instead of the wholesome, nutritious food.

The whole grain does not necessarily include the husk or bran; and in some cases this may be objectionable—not on account of its color, but because of its roughness. Still, the wheat meal containing the bran, in the form of Graham flour, is useful in some cases of constipation, and especially when the food has consisted principally of concentrated articles, such as fine flour biscuits, light bread, cakes, etc.

So far as color is concerned, it is a strange perversion of taste to prefer the dull white color of bolted flour, to the beautiful golden or bronze color of whole wheat flour, if the product of good sound wheat. This color may vary somewhat, according to the kind of wheat, but any sound wheat meal, whether from the white or red variety, is more attractive in appearance to an unperverted eye, than the white or bolted flour. Let it be remembered, always, that the color is due to the nutritive properties of the grain, and that just so far as it is deprived of this color, to that extent its value as a food is diminished. In this coloring matter is contained the phosphates which are essential to the growth and nutrition of the body in general, and to the bony and nervous systems in particular.

And yet in the face of these incontrovertible facts, people persist in using fine bolted flour; instead of appropriating the food prepared for them by nature in the most attractive and palatable form, they go to the doctor's, or drug stores, and are dosed with expensive, and often useless compounds in the way of vitalized phosphates, etc. Mothers who pursue such a suicidal course, not only destroy their own health, but also inflict irrreparable injury on their offspring by depriving them of the natural food provided for building up their bones, muscles, brain and nerves. While such a mode of living is persisted in, the disastrous effects can never be counteracted by all the doctors and drug stores in the world.

Finally, let it not be forgotten that whole wheat bread is about four times more nourishing than any kind of meat.

